# Phosphatidylcholine and its relation to apolipoproteins A-1 and B changes after Roux-en-Y gastric bypass: a cohort study

**DOI:** 10.1186/s12944-019-1111-7

**Published:** 2019-09-05

**Authors:** Elin Rebecka Carlsson, Kristine H. Allin, Sten Madsbad, Mogens Fenger

**Affiliations:** 1 Department of Clinical Biochemistry, Copenhagen University Hospital Hvidovre, Hvidovre, Denmark; 2 Center for Clinical Research and Prevention, Bispebjerg and Frederiksberg Hospital, The Capital Region, Copenhagen, Denmark; 3 Department of Endocrinology, Copenhagen University Hospital Hvidovre, Hvidovre, Denmark

**Keywords:** Phospholipids, Obesity, Gastric bypass surgery, Diabetes, Phosphatidylcholine, Apolipoproteins, Human, Clinical research

## Abstract

**Background:**

Phosphatidylcholine (PC), the most abundant of the phospholipids, has several metabolic functions in organs such as the liver and the intestine, important structural- and signaling functions in biological membranes, and might have a role in the effects of Roux-en-Y gastric bypass (RYGB), an operation known to ameliorate metabolic diseases, including type 2 diabetes. We hypothesized that serum PC, as a reflection of phospholipid metabolism, changes after RYGB, and that changes are related to weight loss and possibly to changes in glucose metabolism (reflected in the HbA1c-level) as well as to changes in serum Apo A1, Apo B and Apo B/Apo A1 ratio.

**Methods:**

In a cohort of 220 RYGB patients, we studied changes in serum PC after RYGB in relation to serum Apo A1 and Apo B, the main apolipoproteins in HDL- and LDL/VLDL-particles, respectively, up to 2 years following RYGB-surgery.

**Results:**

Serum PC reached its lowest levels 3 months postoperatively to later rebound to preoperative levels 24 months after RYGB. No difference was seen between patients with or without type 2 diabetes. Serum Apo A1 showed a similar pattern whereas serum Apo B concentrations stayed low after the initial decrease after RYGB. As a result, the Apo B / Apo A1 ratio constantly decreased during follow-up. There was a strong positive correlation between PC and Apo A1, and between PC and Apo B, but none between Apo A1 and Apo B. After RYGB surgery, both PC and Apo A1, but not Apo B, correlated positively to weight loss. In relation to total cholesterol, the molar ratio between serum PC and plasma cholesterol increased steadily after RYGB.

**Conclusions:**

We conclude that changes in PC and apolipoproteins after RYGB are highly dynamic, reflecting a large plasticity and capability of accommodating lipid metabolism including PC-, cholesterol- and apolipoprotein metabolism imposed by RYGB surgery, independent of glucose tolerance. We suggest that after RYGB and major weight loss, PC and Apo A1 might have a special role in the altered metabolism of lipoproteins.

**Electronic supplementary material:**

The online version of this article (10.1186/s12944-019-1111-7) contains supplementary material, which is available to authorized users.

## Background

Within the research field of metabolic disease, increased attention has been given to lipids in biological membranes. For instance, major glycerophospholipids phosphatidylcholine (PC) and phosphatidylethanolamine, are suggested to be involved in development and progression of metabolic disease [1], as membrane components and perhaps as ligands to nuclear receptors [2].

Recently, we described how levels of sphingomyelin, another membrane lipid, related to diabetes status in an obese population operated with Roux-en-Y gastric bypass (RYGB) [3]. The RYGB-operation has been catching interest mainly because of its large effects on weight and glucose homeostasis in the short- and long term [4, 5]. Still, the underlying formula to its success is not fully understood [6] but might involve factors like changes in sphingo- and phospholipids.

PC is the most abundant phospholipid [1] with its highest concentrations found in high density lipoprotein (HDL) particles [7]. In all mammalian cells, PC is synthesized from choline, which after entering the cell, passes through steps of phosphorylation and conformational changes before finally reacting with diacylglycerol in the endoplasmatic reticulum to generate PC [1]. PC has several important metabolic functions in organs such as the liver [1, 8, 9] and the intestine [1, 10]. For instance, PC seems to play a role in intestinal secretion of lipoprotein particles in the form of chylomicrons. Apart from this, it is also involved in uptake of fatty acids, which can affect the release of glucagon-like peptid-1 (GLP-1) from L-cells in the distal small intestine [1]. Recently, it has been shown that intestinal de novo PC synthesis via CTP:phosphocholine cytidyltransferase-α is required for dietary lipid absorption when on a high fat diet [10]. Various PCs are involved in lipid metabolism as ligands for several of the nuclear transcription factors belonging to the Peroxisome Proliferator-Activated Receptor family (PPARs). Also, saturated PC may regulate glucose metabolism as a substrate provider to diglyceride kinases delta that may be causative in insulin resistance (see Furse and colleagues [9] for a detailed account of PC’s almost ubiquitous role in metabolism). However, the potential use of PC levels in a clinical setting remains to be determined.

After RYGB, a decrease in PC levels as well as increased levels of PC, depending on the PC-subspecies have been reported [11–14]. Graessler and others, for example, observed a reduction in total plasma PC and in 14 of 33 PC metabolites 3 months after RYGB in five morbidly obese subjects, where four had T2D [11]. In a study by Kayser and others that compared changes after RYGB with changes after adjustable gastric banding 1 and 3 months after surgery in a total of 59 obese women, changes in serum PC were related to surgical technique and for PC species with less than 36 carbons a more sustained decrease was seen in the RYGB group [12]. Arora and others found that plasma levels of single PC subspecies correlated to insulin levels in 16 obese patients (14 with T2D) 4 and 42 days after RYGB. Also, pre-surgery plasma levels of single PC species were associated with remission of T2D in this study [13]. Lastly, 12 months after RYGB, PC plasma concentrations were increased in ten individuals with T2D in a study by Lopes and others [14].

Here, we describe changes in serum PC in relation to its main lipoprotein captains of transportation, that is, Apolipoprotein A1 (Apo A1) in HDL and Apolipoprotein B (Apo B) in LDL, after RYGB at 3, 6, 12 and 24 months follow up in a large patient cohort (*n* = 220). Levels of Apo A1 and Apo B have both previously been shown to change after RYGB [15–18]. We hypothesized that serum PC, as a reflection of phospholipid metabolism, changes after RYGB, and that changes are related to weight loss and possibly to changes in glucose metabolism (reflected in the HbA1c-level) as well as to changes in serum Apo A1, Apo B and Apo B/Apo A1 ratio. In addition, as the measurement of serum free choline was a part of the PC-assay and that availability of choline might affect PC metabolism [19], results from measurement of free choline will be presented.

## Methods

### Research- and reference populations

The research population of 220 obese patients, treated with RYGB-surgery at Copenhagen University Hospital Hvidovre between November 2010 and September 2013 has been previously described [3, 20]. In brief, all 220 included patients had delivered a fasting serum blood sample before their operation and another sample within 4 months after surgery. Many of the patients continued to deliver samples during postoperative follow-up, and a total of 889 samples from up to 2 years after RYGB could be included in this study. Samples were binned in the categories 3, 6, 12 and 24 months after RYGB as previously described [3]. All samples were frozen shortly after sampling at − 80 °C and stored between 9 months and 5 years at the time for measurement of serum PC and between 3 and 7 years at the time for measurement of apolipoproteins A1 and B.

Subdivision of the research population in subgroups according to diabetes status has been previously described [3]. Three main subgroups are addressed in this paper; NDM, a non-diabetes mellitus (DM) group (*n* = 151); DMH-NDM, a group of patients with DM and hyperglycemia who obtained remission of DM after RYGB (*n* = 34); DMH-DMH, a group of patients with DM and hyperglycemia who did not obtain remission after RYGB (*n* = 20). Remission was defined as an HbA1c-decrease to below 48 mmol/mol (6.5%) without any antidiabetic medication for as long as there were available clinical- and laboratory data, varying from 2 years to a maximum of 5 years.

After surgery, patients were instructed to eat according to general international dietary recommendations and common practice after RYGB-surgery [21]. In practice, this meant liquid nutrition the first post-operative week and over the following 2 weeks a gradual return to solid foods. From week four, patients were advised to eat regular protein-rich foods (5000 kJ/1200 kcal and 80 g protein) in small portions, with an approximate relative energy content of 42% carbohydrate, 24% protein and 31% fat (maximally 10% saturated fat, as recommended in the Nordic Nutrition recommendations [22]). From month four and onwards, the dietary plan was individualized by a clinical dietician to meet the specific needs for each patient. When weight was stabilized 1 year after surgery, the recommended daily calorie intake corresponded to energy expenditure for the individual patient.

From a 6784-subject biobank with samples from the general Danish population, we picked 235 serum samples that were used to establish an approximate reference interval for PC. The reference population consisted of female and male subjects without known disease and with approximately the same age distribution as our research population. The samples had been stored at − 80 °C for about 15 years and were all thawed and refrozen a few but an equal number of times.

### Biochemical analysis

Serum PC levels were determined by measuring the choline content of PC with an in-house enzymatic assay that was slightly modified from our recently described sphingomyelin assay [3]. A 10 μL serum sample was incubated with a PC-specific phospholipase D (3 U), choline oxidase (0.7 U) and peroxidase (15 U) in 100 μL of a buffer of 0.05 M Trishydrochloride and 0.66 mM Calcium Chloride (pH 7) with 0.1% Triton X-100, added 2 mM DAOS [N-ethyl-N-(2-hydroxy-3-sulfopropyl)-3,5-dimethox-yaniline] and 0.72 mM 4-aminoantipyrin. The standard solution was choline chloride (30,000 μmol/L diluted with buffer to concentrations between 900 and 3300 μmol/L) and PC (diluted in 2% triton X-100 in ethanol) was used to control assay level. All samples from the individual patients were analyzed at the same time, in double determinations.

Reagents were phospholipase D (T-39) from Asahi Kasei; choline oxidase (037–14,401) from Wako Chemicals GmbH; peroxidase (P6782), 4-aminoantipyrine (A4382), DAOS (E8381), choline chloride (C7017), L-α-phosphatidylcholine (P3556), L-α-lysophosphatidylcholine (L1381) and sphingomyelin (S0756) from Sigma-Aldrich Denmark A/S; Tris Hydrochloride from VWR Life Science; Triton® X-100 and calcium chloride from Merck & co., Inc.

Endpoint absorbances at 595 nm were read spectrophotometrically after 90 min in 37° Celsius by SpectraMax i3x from Molecular Devices on a standard 96 well microplate. Results were calculated with the software SoftMax Pro 6.4 and could be reproduced in double within the same run with a SD of 34 μmol/L and a CV at 1.9%. Intermediary precision SD was between 48 μmol/L (at the level of 1418 μmol/L over 19 independent time points of analysis) and 153 μmol/L (at the level of 2780 μmol/L over 31 independent time points of analysis), and intermediary CV was less than 5%. Linearity was documented by serial dilution of a sample with high concentration from 579 to 2909 μmol/L and recovery was between 94 and 104% when adding PC of known concentration to a sample with an endogenous PC concentration at 1567 μmol/L. The phospholipase was described to be specific to glycerophospholipids, with a relative activity towards PC at 100%, lysophosphatidylcholine at 3.4% and sphingomyelin at 0.03%. We found no interference by sphingomyelin, but for lysophosphatidylcholine there was a recovery of between 57 and 71% when it was added in known concentrations to a sample.

Because endogenous free choline and possibly free hydrogen peroxide can interfere with the assay principle, we repeated the analysis with a lower set of standards and without phospholipase, as previously described [3]. As the levels of free choline were less than 5% of PC concentrations in the serum samples from the study population, we did not adjust for this interference, but have chosen to report these results independently as a measure of “free choline”. This part of the assay correlated well when we compared our measures of serum free choline to measurements of serum choline with a commercial assay from Sigma Aldrich (MAK056) [*r* (39) = 0.912, *p* < 2e^− 16^], but our concentrations were generally about 2.5 times higher. In a subsample of our material we also measured hydrogen peroxide and glutathione with commercial assays from Cell Biolabs, Inc. (STA-344) and Thermo Fisher Scientific (EIAGSHC), respectively. The concentrations of both analytes were very low and were not assumed to influence the measure of PC significantly. The size of the interference was higher (up to 16%) in the samples from the reference population, possibly due to differences in handling and storage conditions. Concentrations of PC in these samples were therefore adjusted according to the measures of free choline.

Serum Apo A1 and Apo B were measured with the immunoturbidimetric Tina-quant version 2 assays for Apolipoprotein A-1 and Apolipoprotein B respectively, on Cobas 6000 c501 module from Roche Diagnostics. The concentrations of Apo A1 and Apo B are reported in μmol/L.

The methods for other routine measures such as HbA1c, Plasma Triglyceride, Plasma Total-, LDL-, HDL- and VLDL cholesterol have been described previously [20].

### Statistical analysis

The IBM SPSS version 22 was used for all analyses. Normal distribution and homogeneity of variances of most parameters generally allowed for use of parametric tests, including the one-way ANOVA and Tukey post hoc test for multiple comparisons across groups, the independent t-test for comparing only two subgroups and the paired t-test for comparing the same group of patients before and after RYGB. To avoid impact of missing data, repeated paired t-tests have been used rather than a repeated measures ANOVA to detect changes following RYGB. Where Levene’s test of equality of variances was statistically significant, the one-way ANOVA was performed with a Welch-Satterthwaite correction followed by a Games-Howell post hoc test. For a few parameters without normal distribution, significant differences between subgroups found using the normal one-way ANOVA were confirmed with a Kruskal-Wallis H-test. This applied to Triglyceride, VLDL-cholesterol and HbA1c. The correlations between lipids-, lipoproteins and body weight were determined by Spearman’s correlation. *P*-values lower than 0.05 were considered statistically significant. Throughout the article, data are written as mean with a 95% confidence interval and correlations as correlation coefficients r_s_, with the degree of freedom and *p*-value*.*

## Results

The preoperative clinical characteristics for all patients, the three main patient subgroups and the reference population are shown in Table 1. Apart from body weight, BMI, and lipid concentrations the study population was comparable to the reference population in most variables. In the study population, patients with diabetes had a higher age than patients without diabetes. Also, patients with diabetes had lower total- and LDL-cholesterol levels. No significant differences were found between the two subgroups of patients with diabetes before surgery. Body weight and BMI were similar in all three subgroups before surgery (mean BMI 42.3 (41.6–43.1) kg/m^2^) as well as at all time points after RYGB (24 months BMI: 29.7 (28.3–31.2) kg/m^2^) [3], which is shown in more detail in Table 2, together with data for absolute and relative weight loss and in Additional file 1: Figure S1A & B. Female and male patients were comparable before surgery on all major clinical parameters apart from height, as previously described [3, 20]. Statin treatment was more frequent in the two subgroups with diabetes patients compared to the subgroup without diabetes (Table 1).

**Table 1:** Preoperative clinical characteristics for all patients and patients grouped according to diabetes status, alongside a normal weight reference population

	All patients^a^ (*n* = 220)	NDM (*n* = 151)	DMH-NDM (*n* = 34)	DMH-DMH (*n* = 20)	ANOVA *p*-value^b^	Reference (*n* = 235)	T-test *p*-value^c^
	mean (SD)	mean (SD)	mean (SD)	mean (SD)		mean (SD)	
Age (years)	44.6 (9.5)	42.1 (9.0)	50.5 (8.1)*	51.5 (7.4)*	7 e^−9^	45.5 (8.9)*	2 e^−4^
Gender (female/male)	150/70	113/38	18/16	9/11	0.01	120/115	1 e^−6^
Height (cm)	171.8 (9.6)	171.0 (9.8)	174.5 (8.0)	171.2 (11.4)	0.167	171.0 (9.4)	0.996
Weight (kg)	125.2 (21.6)	126.3 (22.4)	126.4 (20.6)	117.6 (19.7)	0.244	79.2 (15.5)*	1 e^−61^
BMI (kg/m^2^)	42.3 (5.8)	43.1 (5.9)	41.4 (5.4)	40.0 (3.6)*	0.006	27.0 (4.5)*	1 e^−81^
Systolic blood pressure (mmHg)	128 (14.7)	126.8 (14.9)	131.2 (12.6)	128.2 (14.7)	0.285	132.1 (19.1)*	0.004
Diastolic blood pressure (mmHg)	82.2 (10.1)	82.0 (11.0)	81.4 (6.8)	82.4 (10.1)	0.93	82.3 (16.7)	0.831
HbA1c (mmol/mol)	39.0 (9.7)	34.5 (3.8)	48.8 (11.7)*	55.2 (10.0)*	4 e^−12^	40.7 (5.8)*	5 e^−26^
HbA1c (%)	5.7 (0.89)	5.3 (0.35)	6.6 (1.07)	6.9 (0.91)	ND	5.9 (0.53)	ND
Total cholesterol (mmol/L)	4.74 (1.04)	4.98 (0.96)	4.22 (1.11)*	4.24 (1.17)*	4 e^−5^	5.54 (1.07)*	5 e^−7^
HDL-Cholesterol (mmol/L)	1.15 (0.30)	1.19 (0.29)	1.06 0.36)	1.09 (0.35)	0.038	1.35 (0.32)*	2 e^−6^
LDL-Cholesterol (mmol/L)	2.88 (0.95)	3.12 (0.85)	2.36 (0.97)* (*n* = 32)	2.27 (1.13)* (*n* = 18)	1 e^−6^	3.69 0.97) (*n* = 67)*	2 e^−5^
VLDL-Cholesterol (mmol/L)	0.71 (0.32)	0.67 (0.30)	0.75 (0.30) (*n* = 32)	0.89 (0.47) (*n* = 18)	0.086	0.58 (0.25) (*n* = 67)*	0.033
Triglycerides (mmol/L)	1.67 (1.09)	1.50 (0.74)	2.11 (1.95)	2.13 (1.26)	0.038	1.28 (0.83)*	0.008
Statin treatment (+/−)	49/171	12/139	17/17*	13/7*	5 e^−7^	NA	

Data are reported as mean (SD). If the number of patients with available clinical data was less than 95% of the total of patients in the group, the actual number is specified. *SD* Standard deviation, *BMI* Body mass index, *HbA1c* Glycated hemogobin, *HDL* High-density lipoprotein, *LDL* Low-density lipoprotein, *VLDL* Very low-density lipoprotein, *NDM* Patients without diabetes mellitus (DM), *DMH-NDM* Patients with DM in remission after Roux-en-y gastric bypass surgery (RYGB), *DMH-DMH* Patients with DM not in remission after RYGB. Reference, a population of healthy individuals with normal weight. ^a^ All patients also include 15 patients who belong to other subgroups than the three showed in table; ^b^ *p*-value from One-way ANOVA comparing the three patient-subgroup means; ^c^ *p*-value from independent samples t-test comparing the reference group mean to the mean in the patient subgroup NDM. ^*^ indicates significant difference (*p* < 0,05) when compared to the NDM group. No significant differences were found between the two diabetes subgroups. The two diabetes subgroups were not compared to the reference group. Post hoc *p*-values from Tukey and Games-Howell are not shown in table. Data on statin treatment were not available (NA) for the reference population

**Table 2:** Post-operative weight data for all patients and patients grouped according to diabetes status

	All		NDM		DMH-DMH		DMH-NDM		ANOVA *p*-value
	N	Mean (95% CI)	N	Mean (95% CI)	N	Mean (95% CI)	N	Mean (95% CI)	
3 months after RYGB									
Weight (kg)	168	102.6 (99.7–105.4)	116	102.9 (99.4–106.4)	14	98.4 (88.3–108.6)	26	103.7 (95.9–111.5)	0.671
BMI (kg/m^2^)	168	34.7 (34.0–35.5)	116	35.2 (34.3–36.2)	14	32.4 (30.5–34.2)	26	34.3 (32.1–36.4)	0.112
Weight loss (kg)	168	22.3 (21.2–23.4)	116	22.6 (21.3–23.9)	14	23.4 (19.3–27.5)	26	21.8 (18.8–24.9)	0.797
Weight loss (%)	168	17.8 (17.1–18.5)	116	17.9 (17.1–18.7)	14	19.1 (16.7–21.4)	26	17.5 (15.4–19.5)	0.556
6 months after RYGB									
Weight (kg)	152	94.5 (91.7–97.4)	104	95.0 (91.5–98.6)	15	92.1 (82.4–101.9)	23	93.0 (85.0–100.9)	0.782
BMI (kg/m^2^)	152	32.1 (31.3–32.8)	104	32.6 (31.6–33.5)	15	30.4 (28.5–32.3)	23	31.1 (28.8–33.4)	0.155
Weight loss (kg)	152	30.5 (28.9–32.0)	104	31.2 (29.4–33.0)	15	28.2 (24.1–32.3)	23	29.7 (25.2–34.1)	0.442
Weight loss (%)	152	24.3 (23.3–25.2)	104	24.7 (23.6–25.7)	15	23.4 (21.0–25.8)	23	24.2 (21.1–27.3)	0.724
12 months after RYGB									
Weight (kg)	115	86.8 (83.7–90.0)	82	85.5 (81.8–89.2)	14	87.6 (76.6–98.7)	14	91.7 (81.9–101.4)	0.448
BMI (kg/m^2^)	115	29.8 (28.9–30.6)	82	29.6 (28.6–30.7)	14	29.0 (26.8–31.3)	14	30.6 (27.8 (33.5)	0.664
Weight loss (kg)	115	35.5 (33.2–37.8)	82	36.7 (34.1–39.3)	14	31.7 (26.9–36.6)	14	32.8 (25.0–40.7)	0.231
Weight loss (%)	115	29.0 (27.4–30.5)	82	30.0 (28.2–31.7)	14	26.7 (23.4–30.0)	14	26.3 (20.7–31.8)	0.139
24 months after RYGB						Mean (min - max)			
Weight (kg)	38	87.2 (81.6–92.8)	26	82.6 (77.0–88.3)	2	69.6 (60.7–78.4)*	7	99.9 (83.2–116.6)	0.013
BMI (kg/m^2^)	38	29.7 (28.3–31.2)	26	28.8 (27.1–30.5)	2	26.8 (25.9–27.7)	7	32.6 (28.2–36.9)	0.094
Weight loss (kg)	38	35.9 (31.1–40.7)	26	38.4 (32.4–44.4)	2	36.4 (24.0–48.6)	7	30.1 (18.2–42.0)	0.421
Weight loss (%)	38	29.0 (25.7–32.3)	26	31.3 (27.5–35.0)	2	33.3 (28.3–38.3)	7	23.5 (13.8–33.1)	0.146

Data are reported as mean (95% CI), except for 24 months postoperative in the DMH-DMH group, where the numbers in parenthesis resembles minimum and maximum values (*N* = 2). *BMI* Body mass index, *CI* Confidence interval, *NDM* Patients without diabetes mellitus (DM), *DMH-NDM* Patients with DM in remission after Roux-en-y gastric bypass surgery (RYGB), *DMH-DMH* Patients with DM not in remission after RYGB. All patients also include 15 patients who belong to other subgroups than the three showed in table; p-value from One-way ANOVA compares the three patient-subgroup means; * indicates significant difference (*p* < 0,05) when compared to the NDM group. No significant differences were found between the two diabetes subgroups

**Fig. 1:**
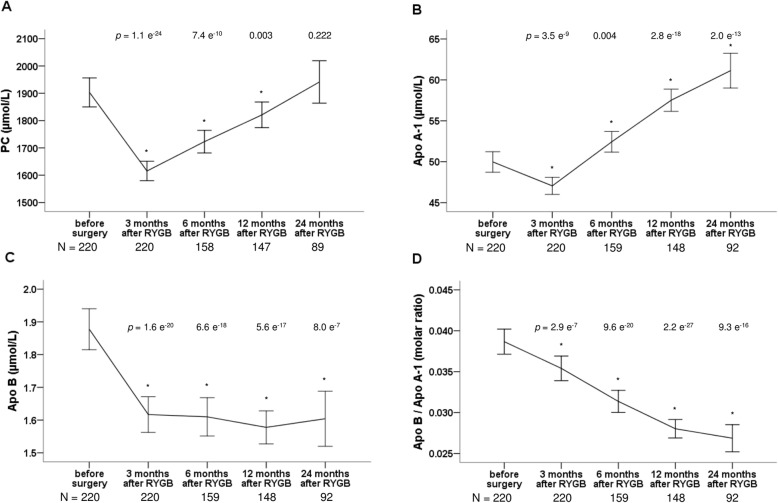
Changes in **a** Phosphatidylcholine (PC), **b** Apolipoprotein A1 (Apo A1), **c** Apolipoprotein B (Apo B) and **d** Apolipoprotin B/A1 molar ratio 3, 6, 12 and 24 months after Roux-en-y gastric bypass surgery (RYGB). Data are shown as means with error bars representing a 95% confidence interval of the mean. * marks a significant different value compared with corresponding preoperative value. *P*-values are from paired t-tests, comparing values after surgery with the corresponding value before surgery. Number of patients (N) at each timepoint is shown at the bottom of each figure

All 220 patients were represented with PC measurements at 3 months follow up and then dropped to 158, 147 and 89 at 6, 12 and 24 months, respectively (Fig. 1a). For Apo A1 and Apo B, numbers were of similar size (Fig. 1b-d). Fifty-five patients were represented at all five timepoints and for 196 and 165 of the patients, PC data were available 6 months after surgery or later and 12 months after surgery or later, respectively.

**Fig. 2:**
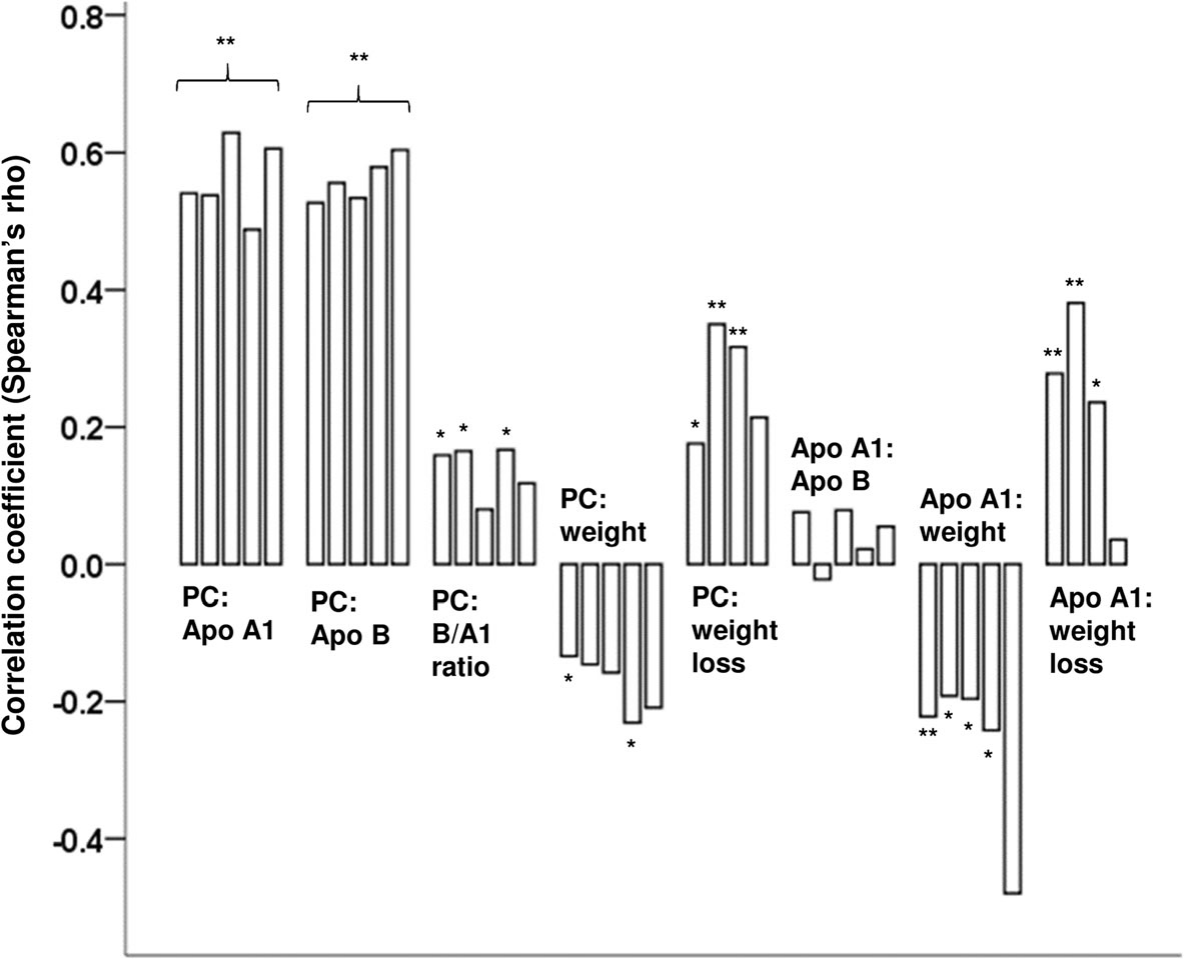
Correlations between PC, apolipoproteins, weight and weight loss and correlations between Apo A1 and Apo B, weight and weight loss. Starting from the left, bars represent correlation coefficients before surgery, 3, 6, 12 and 24 months after Roux-en-y gastric bypass (RYGB) surgery, respectively. For correlations to weight loss, the first bar is the correlation 3 months after RYGB. Significant correlations are shown as * (*p* < 0.05) and ** (*p* < 0.01). Correlations between PC and Apo A1 and B respectively, were all significant on the 0.01-level

Comparing serum PC concentrations, mean levels fell from 1903 (1850–1956) μmol/L before surgery to 1615 (1580–1651) μmol/L 3 months after RYGB (*p* = 1.1 e^− 24^) in the whole study population (Fig. 1a). Over the next 21 months of follow up, we observed a gradual increase towards pre-surgical values. The same trend was seen in all patient subgroups (Additional file 2: Figure S2A). Female patients had higher levels than male; 1954 (1885–2023) μmol/L compared to 1794 (1719–1868) μmol/L before surgery (*p* = 0.005) and 1651 (1611–1690) μmol/L compared to 1540 (1468–1612) μmol/L 3 months after RYGB (*p* = 0.004). As shown in a supplementary table, no differences between patients with or without diabetes nor between the two diabetes subgroups were evident (Additional file 3: Table S1) and, in addition, there was no correlation between PC and HbA1c levels. Serum levels of PC showed a moderate positive correlation to weight loss, that was highly statistically significant within the first 12 months after surgery (Fig. 2). The correlation of serum PC to body weight was not very strong (Fig. 2) and no correlation was detected to BMI.

In the reference population, mean serum PC concentration was 1956 (1910–2003) μmol/L. In this population, the gender difference was smaller than in the study population and not significant. There were no correlations between PC and body weight, nor between PC and BMI in this population.

The serum Apo A1 concentration decreased after RYGB from 49.97 (48.72–51.23) μmol/L to 47.05 (46.00–48.09) μmol/L 3 months after RYGB (*p* = 3,5 e^− 9^) (Fig. 1b). However, 6 months after RYGB levels had increased above pre-surgery levels (*p* = 0.004) and continued to increase significantly during the remaining follow up period to a mean concentration of 61.13 (58.98–63.28) μmol/L at 24 months postoperatively (Fig. 1b). The same trend, shown in detail in a supplementary figure, was seen in all patient subgroups (Additional file 2: Figure S2B). A gender difference in Apo A1 levels was observed before as well as after surgery as the levels for female patients, shown in more detail in a supplementary table, were approximately 5–10% higher than levels for male patients (Additional file 4: Table S2).

For patients with and without diabetes, serum Apo A1 levels were comparable and there were no differences between the two diabetes subgroups (Additional file 3: Table S1). There was a strong positive correlation between serum PC and Apo A1 at all timepoints and similarly to PC, Apo A1 also correlated positively to body weight loss and negatively to body weight (Fig. 2), but not to BMI.

For Apo B, changes in serum concentrations occurred immediately after RYGB, where a significant fall was observed from 1.88 (1.81–1.94) μmol/L before surgery to 1.62 (1.56–1.67) μmol/L 3 months after RYGB, *p* = 1.6 e^− 20^ (Fig. 1c) in the study population. Thereafter, levels were stabilized at this lower level (Fig. 1c). Trends were similar in all subgroups (Additional file 2: Figure S2C). Apo B levels were marginally higher for female patients compared to male patients before surgery and at 3 months after RYGB, details are shown in Additional file 4: Table S2. Apo B concentrations differed significantly between patients with and without diabetes both before surgery and 3 months after RYGB, where patients with diabetes had lower mean levels (Additional file 3: Table S1). The difference was no longer statistically significant at 6, 12 and 24 months. Between the two diabetes subgroups, there was a significant difference in Apo B levels 3 months after RYGB, with lower levels in the DMH-DMH group than in the DMH-NDM group (Additional file 3: Table S1). From this point and onwards, levels in the DMH-NDM group did no longer differ significantly from levels in the NDM group. Just as for Apo A1, there was a strong positive correlation between serum PC and Apo B at all timepoints, but no correlation between serum Apo A1 and Apo B (Fig. 2). Except for a weak correlation to BMI before surgery (r_s_ (218) = 0,141, *p* = 0.037), serum Apo B did not correlate to body weight, BMI or weight loss.

The ratio between serum Apo B and Apo A (Apo B / Apo A1) decreased after RYGB in a linear manner in the whole study population (Fig. 1d) and in all three subgroups. This is shown in Additional file 2: Figure S2D. Before surgery, patients with diabetes had lower values than patients without diabetes (0.034 (0.031–0.037) compared to 0.041 (0.039–0.042), *p* = 2 e^− 4^). Three months after RYGB, only the subgroup of diabetes patients with persistent hyperglycemia had lower ratios compared to the patients without diabetes and there was a significant difference between the two diabetes subgroups (Additional file 3: Table S1). No gender differences were observed for the Apo B / Apo A1 ratios. A weak positive correlation was seen between serum PC and the Apo B / Apo A1 ratio before surgery and at 3 and 12 months after RYGB (Fig. 2).

**Fig. 3:**
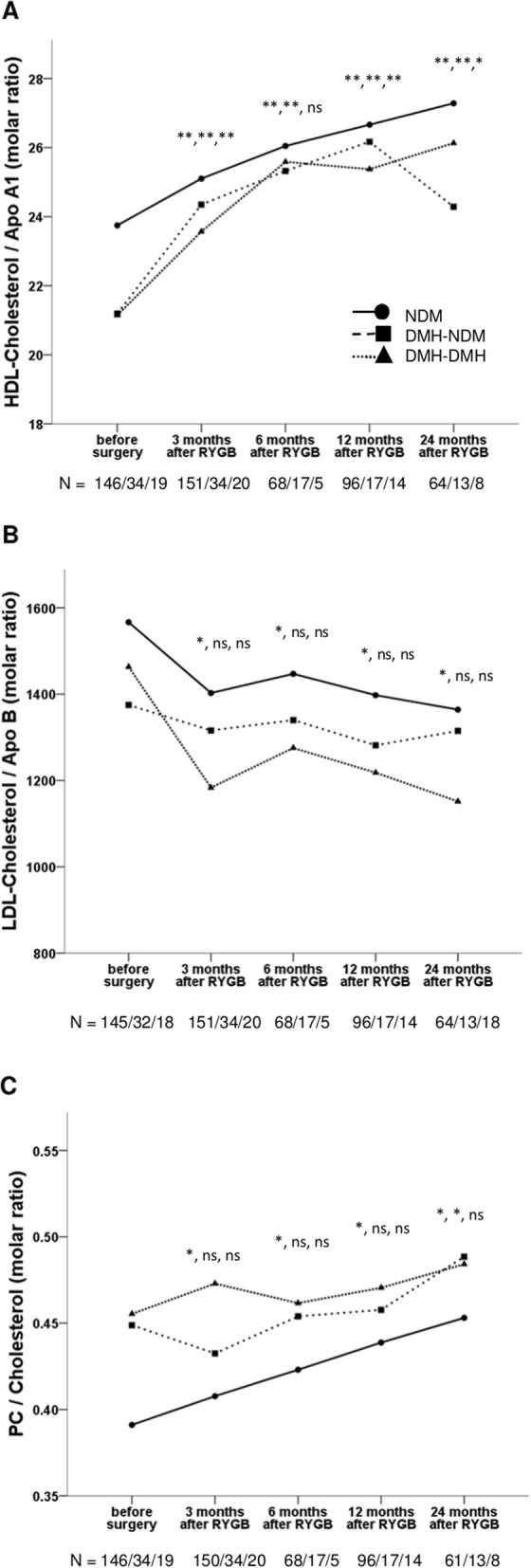
Changes in **a** HDL-cholesterol / Apo A1 molar ratio, **b** LDL-cholesterol / Apo B molar ratio and **c** PC / Cholesterol molar ratio at 3, 6, 12 and 24 months after Roux-en-y gastric bypass (RYGB) surgery in three patient subgroups: NDM, patients without diabetes mellitus (DM) (solid line and circles); DMH-NDM, patients with DM in remission after RYGB (dashed line and boxes) and DMH-DMH, patients with DM and continued hyperglycemia after RYGB (dotted line and triangles). Data are shown as mean. Significant different values compared with corresponding preoperative value are shown as * (*p* < 0.05), ** (*p* < 0.01) and ns (not significant) in the order NDM, DMH-NDM, DMH-DMH. The number of patients (N) for whom we have data at each time point are also shown in the order NDM/DMH-NDM/DMH-DMH at the bottom of each figure

As expected from changes in their respective concentrations in serum, the ratio of PC to Apo A1 decreased after RYGB, which was a change in opposite direction compared to the ratio of PC to Apo B. Changes in the ratio of PC to Apo B were however small and not statistically significant in the subgroups with patients with diabetes. The ratios of plasma total cholesterol to serum Apo A1 and Apo B followed the same pattern as the corresponding serum PC-apolipoprotein-ratios. Interestingly, the ratio of HDL-cholesterol to Apo A1 increased from 23.0 (22.5–23.5) μmol/μmol before surgery to 24.9 (24.4–25.3) μmol/ μmol 3 months after RYGB, *p* = 2.5 e^− 16^ and continued to increase during the rest of the follow up period in all patient subgroups (Fig. 3a). The opposite trend was seen for the ratio of LDL-cholesterol to Apo B (Fig. 3b) but was only statistically significant in the subgroup of patients without diabetes. At all timepoints except 6 months after RYGB, female patients had significantly higher ratios of HDL-cholesterol to Apo A1 than male patients. There was no gender difference for the ratio of LDL-cholesterol to Apo B.

In relation to plasma total cholesterol, the PC / cholesterol molar ratio differed significantly between patients with and without diabetes before surgery, 0.392 (0.382–0.402) compared to 0.453 (0.425–0.481) (*p* = 1.4 e^− 4^) and increased linearly after RYGB in the group of patients without diabetes (Fig. 3c). At all timepoints, the PC / cholesterol molar ratios where the same both for female and male patients.

The influence of statin treatment on lipid- and lipoprotein concentrations are summarized in Additional file 5: Table S3. Only subtle differences were seen between patients without diabetes using statins or not. For patients with diabetes, serum LDL-cholesterol concentrations were lower among statin users. Serum Apo B, and to some extent the Apo B / Apo A1 ratio, were also lower in the statin treated group. Serum PC levels did not differ, statin treatment or not, for patients both with and without diabetes.

**Fig. 4:**
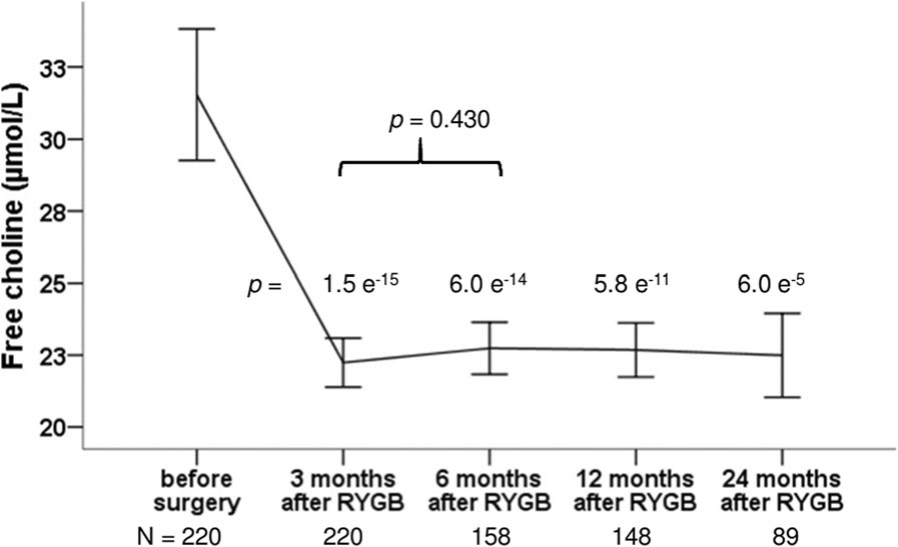
Changes in free choline at 3, 6, 12 and 24 months after Roux-en-y gastric bypass surgery (RYGB). Data are shown as means with error bars representing a 95% confidence interval of the mean. *P*-values are from paired t-tests, comparing values after RYGB with the corresponding value before surgery and values at 6 months after RYGB with values at 3 months after RYGB

Serum concentrations of endogenous free choline decreased immediately after RYGB, from 31.54 (29.26–33.82) μmol/L before surgery to 22.24 (21.39–23.09) μmol/L 3 months after RYGB, *p* = 1.5 e^− 15^ and stayed at the lower level for the rest of follow up period (Fig. 4).

## Discussion

In the present study we report the dynamic development in the major apolipoproteins, cholesterol and PC after RYGB. In the short term, serum Apo A1 and PC exhibited a significant decrease, however, which turned to an increase, approaching the pre-surgery levels after 2 years. Interestingly, the cholesterol content of the Apo A1 particles constantly and robustly increased during the 24 months survey without a dip in levels at 3 months after surgery. The serum levels of Apo B also decreased after RYGB, but in contrast to Apo A1 the levels stayed below pre-surgery levels without any rebound. As a result, the Apo B / Apo A1 ratio constantly decreased during the first 2 years of follow-up after RYGB.

Similar to what Graessler [11] and Kayser [12] reported from their studies that compared plasma PC levels before and after RYGB surgery in obese patients, we observed a decrease in serum PC 3 months after RYGB. The methods used by Graessler and Kayser both allowed for a distinction between PC species which in Kayser’s study revealed that depending on carbon chain length, the metabolites might change in different directions after RYGB [12]. The two studies mentioned above did not report further follow-up after the first three postoperative months, but in another study, Lopes and coworkers looked at long term changes and reported increased levels of PC 12 months after RYGB in a group of patients with diabetes and a BMI between 30 and 35 [14].

The size and direction of correlations between serum PC and Apo A1 and Apo B that we have reported here were similar to those previously reported by Kayser [12].

The changes we observed in serum Apo A1, Apo B and the Apo B / Apo A1 ratio after RYGB were in agreement with results from previous studies [16, 17, 23]. The increase in serum Apo A1 reflects an increase in the number of HDL-particles and in addition, also an increase in the average amount of cholesterol in the HDL-particle was seen after RYGB. The decrease in plasma total cholesterol that we observed after RYGB in our study population was simply caused by the decrease of cholesterol in Apo B containing particles that is a hundred times larger than the corresponding increase in the average HDL-particle.

The linear increase in PC / cholesterol ratio after RYGB and the similar patterns of changes in relation to lipoproteins for both PC and cholesterol might suggest that levels of PC per HDL-particle increased in a similar manner as cholesterol. This is of course highly hypothetical, as this study does not measure HDL-PC levels, but it would agree well with results from Aron-Wisnewsky’s study on 34 morbidly obese women that showed an increase after RYGB in phospholipid content in a sub-fraction of large HDL that itself was statistically significantly increased after RYGB [23]. PC is known to be transported almost exclusively in HDL and LDL particles and not for instance bound to albumin [7].

It is not known whether the changes in serum PC concentration are a result of the altered lipoprotein particle ensemble after RYGB or if PC-metabolism is driving the changes in lipoprotein numbers and composition. In the liver, PC is of general importance for secretion of VLDL particles and can, in addition to the common pathway, be synthesized through methylation of phosphatidylethanolamine [1]. PC, delivered to the liver by HDL or LDL, is also a major source of triglycerides; through the action of phospholipase C, PC is hydrolyzed into diacylglycerol and phosphocholine after which the diacylglycerol is acylated to triglyceride [8]. It is well known, that triglyceride drives the assembly of VLDL and if not secreted, it is involved in development of liver steatosis. Furthermore, PC as a ligand to PPARs in the liver seems to have functions in gene regulation of factors involved in lipid-, bile- and glucose metabolism [9].

In the groups of patients with diabetes, we observed no changes in ratios involving Apo B and or LDL-cholesterol. This might in part be explained by the large heterogeneity in these groups but can also be due to the higher frequency of statin therapy, a frequency that was not constant in the follow up period and that differed between the two diabetes subgroups. Therefore, changes in the group of patients without diabetes probably best resemble the “true” changes after RYGB. Some statins have also been shown to affect phospholipid concentrations [24–26]. In our population, simvastatin is the most used statin, and was not seen to affect serum PC nor Apo A1 levels.

We observed a fall in mean serum choline concentrations after RYGB to a much lower level that was constant during the rest of the follow up period. The supply of intestinal choline may be reduced by the by-pass of the phosphocholine-generating alkaline sphingomyelinase in the proximal intestine [27]. A decrease in choline absorption and/or a decreased intestinal de novo PC synthesis and lipid uptake may at least in part explain the decrease in serum PC in the short-term after RYGB. This is, of course, speculative, as we did not investigate these mechanisms directly. The following increase in serum PC may (just as speculatively) arise from a redistribution of PC from extrahepatical tissues in HDL to sustain the basal delivery of choline to the liver or reshuffling of choline from other sources to PC synthesis.

One important confounder was the variation in time between sampling of the pre-surgical sample and date of surgery, some samples taken months before surgery. This means that some changes that we see might in part be attributable to life style changes before surgery, as for example described by Kjellmo, who reported of a preoperative 10% weight loss and significant decrease in Apo B [16].

Furthermore, we do not have registrations on individual-level dietary intake. The observed changes in PC and apolipoproteins may partly be explained by the changes in total energy intake that generally are seen after RYGB (an initial decrease, followed by a gradual increase towards a level that matches the new weight). Less likely, changes in PC and apolipoproteins may result from changes in food preferences. However, it has previously been shown that food preferences were unchanged 6 months after metabolic surgery in a study-population similar to ours [28]. Studies of intense lifestyle interventions with weight loss following 12 weeks or 12 months diet restriction with or without exercise have found similar changes in total- and cholesterol fractions, Apo A1 and Apo B immediately after the intervention period [29, 30]. It is not known, if these effects can be maintained when the intervention is discontinued. Very similar to our observations and after a comparable weight loss, a longitudinal study on RYGB found a 30% decrease in the ApoB/ApoA1 ratio 2 years after surgery [31]. This improvement was even beyond levels in a matched never-obese normal weight control group, indicating an additional, weight independent effect of RYGB. Similar effects were observed for total- and LDL-cholesterol. A procedure specific effect is also implied by the fact that RYGB, in spite of a comparable weight loss, was superior to sleeve gastrectomy in reversing hyperlipidemia both short and long term [32, 33]. Also, observed early changes in total- and LDL-cholesterol after RYGB, before any substantial weight loss has occurred, further support the hypothesis that some changes in lipid levels are a direct result of surgery [34]. Our study describes the effects on PC and apolipoproteins after RYGB-surgery but does not investigate any specific mechanisms of action of surgery m to for example food restriction or large weight loss.

## Conclusion

In conclusion, the most striking findings in the present study are the dynamic changes in circulating levels of the major apolipoproteins, cholesterol and PC. In the short term after RYGB, serum Apo A1 and PC exhibited a significant decrease, however, which turned to an increase approaching the pre-surgery levels after 2 years. This pattern was most pronounced for patients without diabetes but was also seen for patients with diabetes. The cholesterol content of the Apo A1 particles constantly and robustly increased during the 24 months survey without a dip in levels at 3 months after surgery. The levels of Apo B also decreased after RYGB, but in contrast to Apo A1 the levels stayed below pre-surgery levels without any rebound. As a result, the Apo B / Apo A1 ratio constantly decreased during follow-up. We interpret the findings as reflective of a large plasticity and capability of accommodating lipid metabolism imposed by RYGB. We suggest that after RYGB and major weight loss, PC and Apo A1 might have a special role in the altered metabolism of lipoproteins.

## Additional files

Additional file 1: **Figure S1.** Changes in Weight, BMI and HbA1c at 3, 6, 12 and 24 months after Roux-en-y gastric bypass (RYGB) surgery in patients without diabetes mellitus (DM), patients with DM in remission after RYGB and patients with DM and continued hyperglycemia after RYGB. (PDF 83 kb)

Additional file 2: **Figure S2.** Changes in PC, Apo A1, Apo B and Apo A1 / Apo B molar ratio at 3, 6, 12 and 24 months after Roux-en-y gastric bypass (RYGB) surgery in patients without diabetes mellitus (DM), patients with DM in remission after RYGB and patients with DM and continued hyperglycemia after RYGB. (PDF 101 kb)

Additional file 3: **Table S1.** Serum levels of PC, free choline and apolipoprotein A1 & B before and after Roux-en-y gastric bypass surgery. (PDF 77 kb)

Additional file 4: **Table S2.** Differences in Apo A1 and Apo B concentrations between female and male patients before and after Roux-en-y gastric bypass. (PDF 54 kb)

Additional file 5: **Table S3.** Lipid- and lipoprotein concentrations before and after Roux-en-y gastric bypass surgery for patients with and without diabetes, in treatment or in no treatment with statin. (PDF 75 kb)

## Data Availability

The raw data supporting the conclusions of this manuscript will be made available by the authors, without undue reservation, to any qualified researcher upon reasonable request.

## Abbreviations

Apo A1: Apolipoprotein A1
Apo B: Apolipoprotein B
BMI: Body mass index
DM: Diabetes mellitus
DMH-DMH: Patients with DM not in remission after RYGB
DMH-NDM: Patients with DM in remission after RYGB
HbA1c: Glycated hemoglobin
HDL: High-density lipoprotein
LDL: Low-density lipoprotein
NDM: Patients without diabetes mellitus
PC: Phosphatidylcholine
PPAR: Peroxisome Proliferator-Activated Receptor
RYGB: Roux-en-Y gastric bypass
T2D: Type 2 diabetes
VLDL: Very low-density lipoprotein
