# Supplementary material for: Phosphatidylcholine and its relation to apolipoproteins A-1 and B changes after Roux-en-Y gastric bypass: a cohort study
Source: Lipids Health Dis. 2019 Sep 5;18:169. doi: 10.1186/s12944-019-1111-7 (PMC6729082; doi:10.1186/s12944-019-1111-7)
Supplement: Supplementary file 1 — Figure S1. Changes in Weight, BMI and HbA1c at 3, 6, 12 and 24 months after Roux-en-y gastric bypass (RYGB) surgery in patients without diabetes mellitus (DM), patients with DM in remission after RYGB and patients with DM and continued hyperglycemia after RYGB. (PDF 83 kb) [file 12944_2019_1111_MOESM1_ESM.pdf]

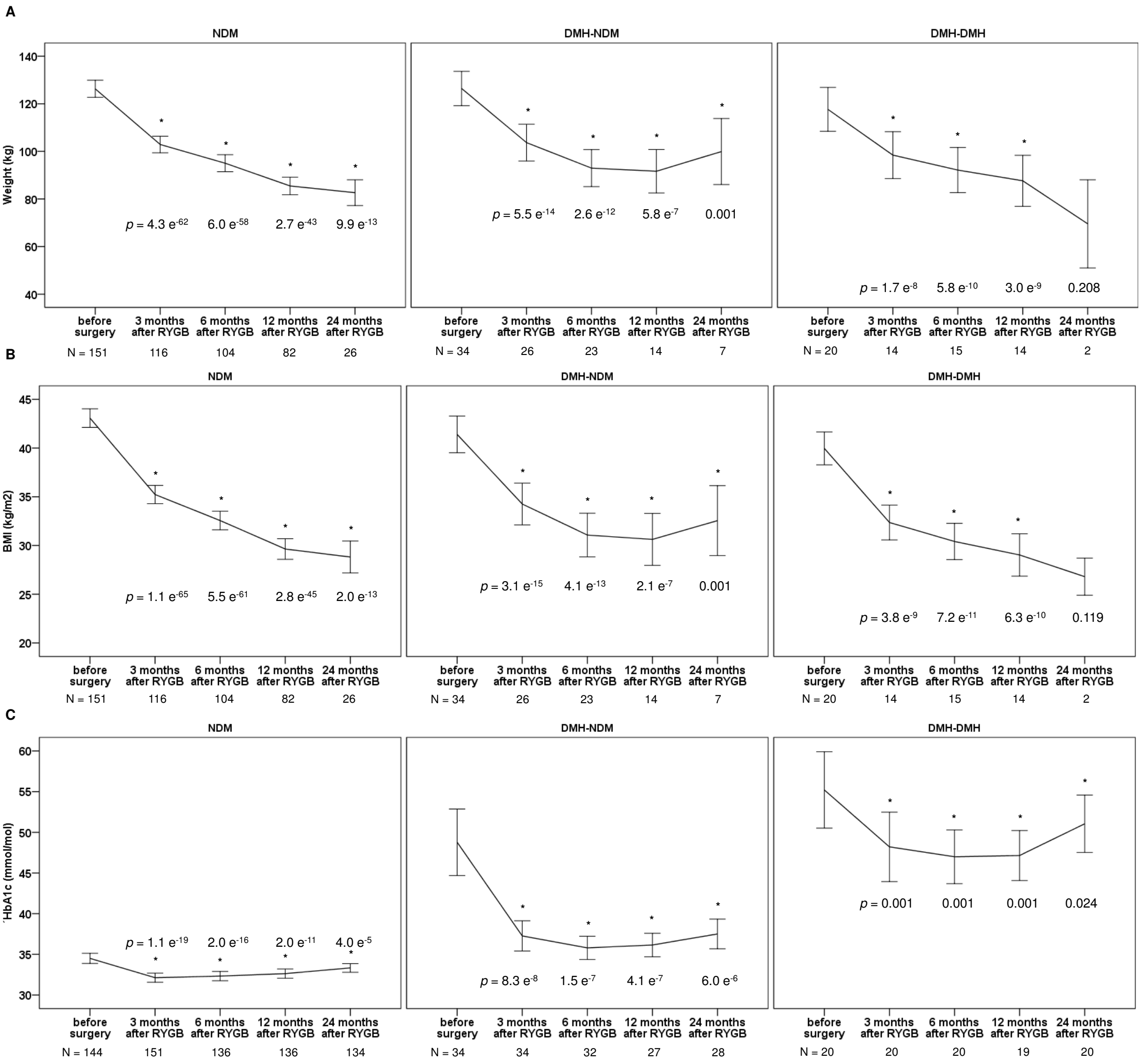

Supplementary Figure 1. Changes in (A) Weight, (B) BMI and (C) HbA1c at 3, 6, 12 and 24 months after Roux-en-y gastric bypass (RYGB) surgery in three patient subgroups: NDM, patients without diabetes mellitus (DM); DMH-NDM, patients with DM in remission after RYGB and DMH-DMH, patients with DM and continued hyperglycemia after RYGB. Data are shown as mean with error bars representing a 95 % confidence interval of the mean. * marks a significant different value compared with corresponding preoperative value. *P* –values from paired t-tests comparing values after RYGB with corresponding values before surgery are shown in each figure, as well as number of patients (N) for whom we have data at each time point.
