# Supplementary material for: Phosphatidylcholine and its relation to apolipoproteins A-1 and B changes after Roux-en-Y gastric bypass: a cohort study
Source: Lipids Health Dis. 2019 Sep 5;18:169. doi: 10.1186/s12944-019-1111-7 (PMC6729082; doi:10.1186/s12944-019-1111-7)
Supplement: Supplementary file 3 — Table S1. Serum levels of PC, free choline and apolipoprotein A1 & B before and after Roux-en-y gastric bypass surgery. (PDF 77 kb) [file 12944_2019_1111_MOESM3_ESM.pdf]

Supplementary Table 1. Serum levels of phosphatidylcholine, free choline and apolipoprotein A1 & B before and after Roux-en-y gastric bypass surgery

	All patients[a] (N=220) mean (95 % CI)	NDM (N=151) mean (95 % CI)	DMH-NDM (N=34) mean (95 % CI)	DMH-DMH (N=20) mean (95 % CI)	ANOVA p-value[b]
**Preoperative**					
Phosphatidylcholine (µmol/L)	1903.1 (1849.9 - 1956.3)	1920.0 (1860.6 - 1979.4)	1856.5 (1669.4 - 2043.6)	1859.5 (1653.8 - 2065.1)	0.628
Free Choline (µmol/L)	31.5 (29.3 - 33.8)	30.9 (28.3 - 33.5)	32.7 (24.7 - 40.7)	34.8 (26.1 - 43.4)	0.609
Apolipoprotein A1 (µmol/L)	49.97 (48.72 - 51.23)	50.04 (48.64 - 51.44)	49.58 (45.30 - 53.86)	50.87 (45.19 - 56.55)	0.895
Apolipoprotein B (µmol/L)	1.88 (1.81 - 1.94)	1.99 (1.92 - 2.06)	1.67 (1.50 - 1.84)*	1.55 (1.35 - 1.74)*	3 e^{-6}
Apolipoprotein B / A1, molar ratio	0.039 (0.037 - 0.040)	0.041 (0.039 - 0.043)	0.035 (0.031 - 0.040)*	0.032 (0.027 - 0.036)*	0.001
**3 months after RYGB**					
Phosphatidylcholine (µmol/L)	1615.4 (1579.7 - 1651.0)	1625.1 (1585.5 - 1664.7)	1615.3 (1513.4 - 1717.2)	1555.1 (1395.5 - 1714.6)	0.678
Free Choline (µmol/L)	22.2 (21.4 - 23.1)	22.1 (21.1 - 23.0)	23.4 (20.7 - 26.1)	21.6 (18.1 - 25.0)	0.472
Apolipoprotein A1 (µmol/L)	47.05 (46.00 - 48.09)	46.91(45.74 - 48.07)	46.63 (43.28 - 49.98)	48.83 (44.08 - 53.58)	0.703
Apolipoprotein B (µmol/L)	1.62 (1.56 - 1.67)	1.67 (1.61 - 1.74)	1.59 (1.43 - 1.74)†	1.33 (1.21 -1.45)* †	2.2 e-5
Apolipoprotein B / A1, molar ratio	0.035 (0.034 - 0.037)	0.037 (0.035 - 0.038)	0.036 (0.031 - 0.041)†	0.028 (0.025 - 0.032)* †	0.001
**6 months after RYGB**					
Phosphatidylcholine (µmol/L)	1722.8 (1681.2 - 1764.4)	1724.6 (1676.2 - 1773.1)	1734.6 (1614.7 - 1854.5)	1661.4 (1421.7 - 1901.2)	0.722
Free Choline (µmol/L)	22.74 (21.83 - 23.65)	22.52 (21.57 - 23.47)	22.76 (20.07 - 25.45)	22.50 (19.87 - 25.13)	0.980
Apolipoprotein A1 (µmol/L)	52.43 (51.17 - 53.70)	52.28 (50.80 - 53.77)	52.56 (48.45 - 56.67)	54.62 (48.78 - 60.46)	0.647
Apolipoprotein B (µmol/L)	1.61 (1.55 - 1.67)	1.65 (1.58 - 1.72)	1.55 (1.36 - 1.74)	1.44 (1.22 - 1.67)	0.148
Apolipoprotein B / A1, molar ratio	0.031 (0.030 - 0.033)	0.032 (0.031 - 0.034)	0.031 (0.026 - 0.036)	0.027 (0.023 - 0.031)	0.047
**12 months after RYGB**					
Phosphatidylcholine (µmol/L)	1821.0 (1773.7 - 1868.3)	1832.6 (1773.2 - 1892.1)	1739.6 (1628.4 - 1850.8)	1731.7 (1575.4 - 1888.1)	0.247
Free Choline (µmol/L)	22.68 (21.74 - 23.63)	22.60 (21.53 - 23.68)	22.0 (18.68 - 25.32)	21.67 (19.07 - 24.26)	0.783
Apolipoprotein A1 (µmol/L)	57.51 (56.14 - 58.88)	58.02 (56.39 - 59.65)	55.69 (50.66 - 60.72)	56.13 (52.06 - 60.20)	0.445
Apolipoprotein B (µmol/L)	1.58 (1.53 - 1.63)	1.59 (1.53 - 1.65)	1.53 (1.35 - 1.71)	1.45 (1.30 - 1.60)	0.214
Apolipoprotein B / A1, molar ratio	0.028 (0.027 - 0.029)	0.028 (0.027 - 0.029)	0.029 (0.024 - 0.034)	0.026 (0.023 - 0.029)	0.436
**24 months after RYGB**					
Phosphatidylcholine (µmol/L)	1941.4 (1862.9 - 2019.9)	1928.9 (1843.11 - 2014.8)	2112.9 (1796.4 - 2429.3)	1745.8 (1542.9 - 1948.7)	0.078
Free Choline (µmol/L)	22.49 (21.02 - 23.97)	21.52 (20.08 - 22.95)	26.64 (20.62 - 32.67)	24.57 (17.69 - 31.45)	0.187
Apolipoprotein A1 (µmol/L)	61.13 (58.98 - 63.28)	60.09 (57.79 - 62.39)	68.70 (60.42 - 76.98)	59.45 (52.35 - 66.55)	0.134
Apolipoprotein B (µmol/L)	1.60 (1.52 - 1.69)	1.62 (1.52 - 1.72)	1.59 (1.28 - 1.90)	1.41 (1.20 - 1.61)	0.417
Apolipoprotein B / A1, molar ratio	0.027 (0.025 - 0.029)	0.027 (0.025 - 0.029)	0.024 (0.019 - 0.029)	0.024 (0.019 - 0.029)	0.228

Data are reported as mean with a 95 % confidence interval of the mean. CI, confidence interval of the mean; RYGB, Roux-en-y gastric bypass surgery.
NDM, patients without diabetes mellitus (DM); DMH-NDM, patients with DM in remission after Roux-en-y gastric bypass surgery (RYGB);
DMH-DMH, patients with DM not in remission after RYGB. N is the number of patients in the group before surgery and at 3 months follow up.
Numbers at other time points can be found in Figures 1, 3 & 4.

[a]All patients also include 15 patients who belong to other subgroups than the three showed in table;

[b] p-value from One-way ANOVA comparing the three patient subgroup means; Post hoc p-values from Tukey and Games-Howell are not shown in table.

* indicates significant difference (p < 0,05) when compared to the NDM group.

† indicates significant difference (p < 0,05) when compared to the other subgroup with patients with diabetes (DMH-NDM or DMH-DMH).
