# Supplementary material for: Phosphatidylcholine and its relation to apolipoproteins A-1 and B changes after Roux-en-Y gastric bypass: a cohort study
Source: Lipids Health Dis. 2019 Sep 5;18:169. doi: 10.1186/s12944-019-1111-7 (PMC6729082; doi:10.1186/s12944-019-1111-7)
Supplement: Supplementary file 4 — Table S2. Differences in Apo A1 and Apo B concentrations between female and male patients before and after Roux-en-y gastric bypass. (PDF 54 kb) [file 12944_2019_1111_MOESM4_ESM.pdf]

Supplementary Table 2. Differences in Apo A1 and Apo B concentrations between female and male patients before and after Roux-en-y gastric bypass.

	Female patients		Male patients		
	N	Mean (95 % CI)	N	Mean (95 % CI)	*p*-value
**Apolipoprotein A1 (µmol/L)**					
Before surgery	150	51.89 (50.25 – 53.53)	70	45.86 (44.43 – 47.28)	$1.1e^{-7}$
3 months after RYGB	150	48.55 (47.24 – 49.87)	70	43.82 (42.34 – 45.30)	$4e^{-6}$
6 months after RYGB	115	53.70 (52.19 – 55.20)	44	49.13 (47.00 – 51.26)	0.001
12 months after RYGB	103	59.05 (57.48 – 60.62)	45	53.98 (51.49 – 56.47)	0.001
24 months after RYGB	66	61.94 (59.60 – 64.27)	26	59.08 (54.10 – 64.06)	0.237
**Apolipoprotein B (µmol/L)**					
Before surgery	150	1.92 (1.85 – 2.00)	70	1.78 (1.67 – 1.90)	0.041
3 months after RYGB	150	1.66 (1.59 – 1.72)	70	1.53 (1.44 – 1.63)	0.043
6 months after RYGB	115	1.64 (1.57 – 1.71)	44	1.53 (1.43 – 1.63)	0.096
12 months after RYGB	103	1.60 (1.55 – 1.66)	45	1.52 (1.41 – 1.62)	0.110
24 months after RYGB	66	1.59 (1.51 – 1.68)	26	1.63 (1.41 – 1.86)	0.731

Data are reported as mean with a 95 % confidence interval. CI, confidence interval of the mean; RYGB, Roux-en-y gastric bypass.
