# Supplementary material for: Phosphatidylcholine and its relation to apolipoproteins A-1 and B changes after Roux-en-Y gastric bypass: a cohort study
Source: Lipids Health Dis. 2019 Sep 5;18:169. doi: 10.1186/s12944-019-1111-7 (PMC6729082; doi:10.1186/s12944-019-1111-7)
Supplement: Supplementary file 5 — Table S3. Lipid- and lipoprotein concentrations before and after Roux-en-y gastric bypass surgery for patients with and without diabetes, in treatment or in no treatment with statin. (PDF 75 kb) [file 12944_2019_1111_MOESM5_ESM.pdf]

Supplementary Table 3. Lipid- and lipoprotein concentrations before and after Roux-en-y gastric bypass surgery for patients with and without diabetes, in treatment or in no treatment with statin

**HbA1c before surgery**			**< 48 mmol/mol**<br>n = 165					**> 48 mmol/mol**<br>n = 55		
	**n**		**statin (+)**	**no statin (-)**	**p-value**	**n**		**statin (+)**	**no statin (-)**	**p-value**
	+	-	mean (95 % CI)			+	-	mean (95 % CI)		
**before surgery**										
Total-cholesterol (mmol/L)	18	142	5.18 (4.36 - 6.01)	4.88 (4.74 - 5.02)	0.45	31	23	3.98 (3.66 - 4.31)	4.55 (3.98 - 5.12)	0.084
LDL-cholesterol (mmol/L)	18	141	3.16 (2.39 - 3.94)	3.04 (2.92 - 3.16)	0.753	28	23	2.01 (1.70 - 2.32)	2.69 (2.20 - 3.18)	0.02 ↓
Triglycerides (mmol/L)	18	142	1.93 (1.58 - 2.27)	1.46 1.34 - 1.58)	0.011 ↑	31	23	2.46 (1.68 - 3.24)	1.69 (1.37 - 2.00)	0.067
Phosphatidylcholine (µmol/L)	18	147	2038 (1823 - 2252)	1902 (1844 - 1959)	0.135	31	24	1878 (1672 - 2085)	1844 (1673 - 2014)	0.799
Apolipoprotein A1	18	147	51.41 (46.09 - 56.72)	49.80 (48.46 - 51.13)	0.449	31	24	50.73 (46.78 - 54.67)	49.01 (43.25 - 54.78)	0.605
Apolipoprotein B	18	147	2.04 (1.69 - 2.40)	1.95 (1.89 - 2.02)	0.608	31	24	1.51 (1.38 - 1.65)	1.77 (1.54 - 1.99)	0.053
Apolipoprotein B/A1	18	147	0.040 (0.039 - 0.042)	0.040 (0.039 - 0.042)	0.679	31	24	0.031 (0.028 - 0.034)	0.038 (0.032 - 0.044)	0.045 ↓
**3 months after RYGB**										
Total-cholesterol (mmol/L)	9	156	3.57 (3.02 - 4.11)	4.03 (3.90 - 4.15)	0.085	18	37	3.31 (2.99 - 3.62)	3.87 (3.54 - 4.20)	0.014 ↓
LDL-cholesterol (mmol/L)	9	156	1.82 (1.32 - 2.32)	2.36 (2.25 - 2.47)	0.026 ↓	18	37	1.56 (1.35 - 1.78)	2.17 (1.87 - 2.47)	0.001 ↓
Triglycerides (mmol/L)	9	156	1.14 (0.84 - 1.44)	1.07 (1.02 - 1.13)	0.55	18	37	1.43 (1.03 - 1.82)	1.18 (1.02 - 1.34)	0.158
Phosphatidylcholine (µmol/L)	9	155	1661 (1486 - 1835)	1613 (1574 - 1652)	0.573	18	37	1605 (1426 - 1783)	1611 (1505 - 1717)	0.945
Apolipoprotein A1	9	156	50.09 (43.59 - 56.58)	46.71 (45.58 - 47.83)	0.171	18	37	49.59 (44.60 - 54.59)	46.51 (43.34 - 49.68)	0.272
Apolipoprotein B	9	156	1.42 (1.16 - 1.69)	1.67 (1.60 - 1.73)	0.077	18	37	1.34 (1.20 - 1.48)	1.59 (1.43 - 1.74)	0.016 ↓
Apolipoprotein B/A1	9	156	0.541 (0.394 - 0.688)	0.667 (0.637 - 0.698)	0.058	18	37	0.028 (0.024 - 0.032)	0.036 (0.031 - 0.041)	0.015 ↓
**6 months after RYGB**										
Total-cholesterol (mmol/L)	8	87	4.55 (3.40 - 5.70)	4.12 (3.98 - 4.27)	0.415	15	21	3.63 (3.23 - 4.04)	4.18 (3.78 - 4.58)	0.06 ↓
LDL-cholesterol (mmol/L)	8	87	2.65 (1.71 - 3.59)	2.3 (2.18 - 2.43)	0.419	15	21	1.66 (1.23 - 2.09)	2.31 (1.92 - 2.71)	0.025 ↓
Triglycerides (mmol/L)	8	87	1.18 (0.70 - 1.66)	0.98 (0.89 - 1.06)	0.174	15	21	1.18 (0.92 - 1.45)	1.16 (0.98 - 1.35)	0.879
Phosphatidylcholine (µmol/L)	7	117	1680 (1454 - 1906)	1726 (1680 - 1773)	0.64	14	20	1624 (1465 - 1782)	1788 (1637 - 1938)	0.13
Apolipoprotein A1	7	118	55.46 (47.59 - 63.33)	51.96 (50.54 - 53.39)	0.256	14	20	55.31 (50.43 - 60.18)	52.13 (47.84 - 56.42)	0.312
Apolipoprotein B	7	118	1.61 (1.23 -1.99)	1.64 (1.57 - 1.70)	0.840	14	20	1.40 (1.20 - 1.60)	1.59 (1.40 - 1.78)	0.157
Apolipoprotein B/A1	7	118	0.029 (0.024 - 0.034)	0.032 (0.031 - 0.034)	0.267	14	20	0.026 (0.021 - 0.031)	0.031 (0.027 - 0.036)	0.100
**12 months after RYGB**										
Total-cholesterol (mmol/L)	7	136	4.47 (3.97 - 4.97)	4.16 (4.04 - 4.28)	0.243	12	35	3.58 (3.13 - 4.02)	4.03 (3.77 - 4.28)	0.072
LDL-cholesterol (mmol/L)	7	136	2.41 (1.80 - 3.03)	2.2 (2.11 - 2.29)	0.312	12	35	1.56 (1.27 - 1.85)	2.07 (1.86 - 2.29)	0.011 ↓
Triglycerides (mmol/L)	7	136	1.05 (0.60 - 1.50)	0.95 (0.90 - 1.01)	0.463	12	35	0.99 (0.71 - 1.27)	1.14 (0.95 - 1.34)	0.405
Phosphatidylcholine (µmol/L)	8	104	2006 (1789 - 2222)	1830 (1773 - 1888)	0.106	10	25	1823 (1564 - 2082)	1723 (1634 - 1811)	0.428
Apolipoprotein A1	8	105	58.23 (50.24 - 66.21)	57.99 (56.40 - 59.57)	0.937	10	25	59.66 (53.38 - 65.94)	54.41 (50.81 - 58.01)	0.118
Apolipoprotein B	8	105	1.71 (1.38 - 2.04)	1.59 (1.54 - 1.65)	0.295	10	25	1.45 (1.17 - 1.72)	1.53 (1.40 - 1.65)	0.531
Apolipoprotein B/A1	8	105	0.031 (0.022 - 0.040)	0.028 (0.027 - 0.029)	0.504	10	25	0.024 (0.020 - 0.028)	0.029 (0.025 - 0.033)	0.132

Data are reported as mean (95 % CI); +, statin treatment; -, no statin treatment;
significant higher or lower values in the statin groupcompared to the group with no statin treatment is marked with ↑ and ↓, respectively.
